# Characterization of glycans of CD4+T cells in HAM/TSP

**DOI:** 10.1186/1742-4690-11-S1-O69

**Published:** 2014-01-07

**Authors:** Daisuke Kodama, Kimiko Izumo, Ryuji Kubota, Toshio Matsuzaki, Hiroshi Takashima, Shuji Izumo

**Affiliations:** 1 Molecular Pathology, Center for Chronic Viral Diseases, Kagoshima University Graduate School of Medical and Dental Sciences, Kagoshima-Shi, Japan; 2 Ohkatsu Hospital, Medical Corporation Sanshukai, Kagoshima-Shi, Japan; 3 Department of Neurology, Kagoshima University Graduate School of Medical and Dental Sciences, Kagoshima-Shi, Japan

## 

The outermost structure of cells is galectin-glycan lattice, that is shown to have many roles in cell-cell interactions. Recently, biofilm-like extracellular viral assemblies were shown to mediate HTLV-1 cell-to-cell transmission. Here, we tried to characterize the glycans on the CD4+T cells in HAM. CD4+T cells from four respective cases of HAM, AC(asymptomatic carriers), and NC(negative control) were subjected to lectin array analysis. Similarly, glycans liberated from membrane proteins of CD4+T cells from six respective cases of HAM and NC analyzed by MALDI-TOF MS. We found that UDA(Urtica dioica agglutinin) and STL(Solanum tuberosum lectin (Potato)) lectins, that recognize N-glycan polylactosamine which consist of repeats of the disaccharide betaGal(1-4)betaGlcNAc(1-3), were significantly high in HAM in lectin array analysis. Interestingly, UDA was reported to inhibit cell-to-cell infection in vitro. On the other hand, MALDI-TOF MS analysis found several O-glycans in HAM. Candidates verified in GlycoSuite database were mainly O-glycans attach to MUC1 and Leukosialin(CD43) as carrier proteins. These proteins were reported to play roles in ATL and cell-to-cell infection as well. These glycans and carrier proteins may be therapeutic target of HAM.

